# The Price of Growing Up in a Low-Income Neighborhood: A Scoping Review of Associated Depressive Symptoms and Other Mood Disorders among Children and Adolescents

**DOI:** 10.3390/ijerph20196884

**Published:** 2023-10-05

**Authors:** Bethany M. Wood, Catherine Cubbin, Esmeralda J. Rubalcava Hernandez, Diana M. DiNitto, Shetal Vohra-Gupta, Philip Baiden, Elizabeth J. Mueller

**Affiliations:** 1 School of Social Work, The University of Texas at Arlington, 211 S Cooper St, Arlington, TX 76019, USA; ejr9470@mavs.uta.edu (E.J.R.H.); philip.baiden@uta.edu (P.B.); 2 Steve Hicks School of Social Work, The University of Texas at Austin, 1925 San Jacinto Boulevard, Austin, TX 78712, USA; ccubbin@austin.utexas.edu (C.C.); ddinitto@mail.utexas.edu (D.M.D.); sgupta@austin.utexas.edu (S.V.-G.); 3 School of Architecture, The University of Texas at Austin, 310 Inner Campus Drive, Austin, TX 78712, USA; ejmueller@austin.utexas.edu

**Keywords:** neighborhood poverty, built environment, gentrification, mental health, depressive symptoms, children, social determinants of mental health

## Abstract

Neighborhoods, as built and social environments, have significant implications for mental health. Children raised in high-poverty neighborhoods, who are disproportionately Black, Indigenous, and people of color, have a greater risk of adverse life outcomes. Neighborhood gentrification is also salient when examining mental health outcomes as neighborhood economic contexts shift around a child. This review scopes, describes, synthesizes, and critiques the existing literature on the relationship between neighborhood poverty/gentrification and mood disorder symptoms among children ages 3–17 in the United States (U.S.). Given the history of structural racism in the creation of U.S. neighborhoods, inclusion criteria required that study samples be racially diverse. Following Preferred Reporting Items for Systematic Reviews and Meta-Analyses (PRISMA) guidelines for scoping reviews, seven databases and grey literature were searched; 17 studies were included (total *n* = 122,089). Fourteen studies found significant associations between neighborhood poverty/gentrification and child depression. Three longitudinal studies found significant results suggesting that childhood neighborhood poverty/gentrification may have a lagged effect, with depression emerging later in life. Neighborhood poverty and gentrification require further examination as social determinants of mental health. Researchers should examine neighborhood poverty and gentrification as social determinants of mental health. Policies that reduce neighborhood economic disparities are needed across the U.S.

## 1. Introduction

Individual-level poverty is associated with many negative health outcomes, and neighborhood-level poverty can compound that relationship or directly affect a person’s health. Neighborhood context has been recognized as a major social determinant of child and adolescent health [1,2,3]. Much of the research on this topic has focused on children’s physical health [4,5]. Less attention has been paid to mental health, despite research showing that nearly half of all mental health problems start during adolescence, with more than 75% occurring by age 24 [6]. The World Health Organization has recognized neighborhood deprivation as one of the five key neighborhood/built environmental social determinants of mental health [7]. The prevalence of depression in youth residing in low-income neighborhoods is also increasing [8]. This review scopes the research literature to examine how neighborhood poverty, as well as neighborhood gentrification, impacts child and adolescent depression, depressive symptoms, and mood disorders. 

### 1.1. Literature Review

Neighborhoods constitute built and social environments that have significant implications for health. Children raised in high-poverty neighborhoods have a greater risk of adverse life outcomes compared to children raised in low-poverty neighborhoods [9] and compared to adults who move into a high-poverty neighborhood from a better-resourced neighborhood [10]. Neighborhood poverty is associated with higher mortality, problems with child sleep, child externalizing problems, the incidence of cardiovascular conditions, asthma, and cognitive decline [11,12,13,14], as well as barriers to healthcare [15,16,17,18]. 

Neighborhood poverty is also associated with an increased risk of adverse mental health outcomes [19], but less is known about the mental health impacts of a sudden increase in neighborhood income through neighborhood gentrification. Residents who remain in a gentrifying neighborhood might be negatively impacted due to: (1) fear of displacement, (2) grief of the loss of neighborhood ties, culture, and connections, and (3) increased prevalence of whiteness as normalcy or supremacy [20,21,22], which can also lead to feeling displaced. This concept of loss echoes the seminal studies of Fried [23], where the loss of a home was reported as being comparable to the loss of a loved one for residents who were forced to relocate. Building on Fried’s work, recent research has also drawn attention to how remaining in a gentrifying neighborhood can lead to a loss of place, which may have a similar effect [24]. For displaced Black and Brown residents, the grief of neighborhood loss to white upper-middle-class families can engender psychological distress. The timing of neighborhood gentrification in a person’s life can also have serious consequences. For these reasons, it is important to understand the current research that explores the complex relationship between neighborhood gentrification and mental health, especially for young children at critical development junctures. 

Despite growing racial/ethnic diversity in the United States (U.S.) [25], many neighborhoods remain segregated along racial/ethnic lines. The history of U.S. neighborhoods is closely tied to systemic racism and antediluvian policies and practices, such as Jim Crow laws and redlining [26,27,28], which promoted neighborhood segregation and disinvestment in Black and Brown communities across the country. For example, in the 1930s, the federal Home Owner’s Loan Corporation asked local leaders to create maps rating neighborhoods in terms of the risk posed to lenders related to making loans in these areas. The highest rankings were given to neighborhoods with newly built single-family homes and white residents, while the lowest rating—coded red—was given to communities of color and neighborhoods with older housing stock. This effectively cut off communities of color from access to credit and furthered the deterioration of the housing stock in these neighborhoods [26,27,29].

Policies that the U.S. government intentionally created to promote neighborhood segregation [30] continue to impact Black and Latine children’s health outcomes. Stark intergenerational racial/ethnic disparities for children growing up in low-income neighborhoods persist. For example, among Black children, 62% born between 1955 and 1970 and 67% born between 1985 and 2000 were raised in low-income neighborhoods, compared to 4% and 6% of white children, respectively [9]. 

A 2021 report found that 81% of urban regions were more segregated in 2019 than they were in 1990 and that the neighborhood poverty rate was 21% in segregated communities of color compared to 7% in segregated white neighborhoods [31]. Furthermore, household incomes and home values in white neighborhoods were twice as high as those in segregated communities of color, and 83% of historically redlined neighborhoods (created in the 1930s) remained highly segregated communities of color [31].

#### 1.1.1. Neighborhoods and Mental Health

Deprivation has been identified as one of the five key neighborhood/built environmental social determinants of mental health [7]. In addition, the WHO reports that depression is one of the top ten causes of disease [32] and was the primary reason for almost 50,000 suicides in the U.S. in 2018 [33]. Cutrona and colleagues [34] developed a framework that identifies several neighborhood factors that contribute to depression, including increased stress due to a lack of resources, the compounding effects of deteriorating neighborhoods, and high resident turnover in low-income neighborhoods disrupting the social ties that mitigate the incidence of depression. As life course theory suggests, experiencing inequalities in childhood and adolescence increases the risk of depression later in life [35]; thus, children growing up in low-income neighborhoods may face disparities in mental health throughout their lives. 

The concepts of neighborhood poverty and mental health are complex and intricately tied to individual, social, environmental, and systemic factors. Neighborhood poverty has, however, been operationalized through variables based on location, measures of housing quality, socioeconomic status, poverty trajectory, and other disadvantages [11,14,36,37,38,39]. The complex nature of these interrelationships makes comparison across studies difficult.

#### 1.1.2. Past Reviews 

Galster [40] reviewed studies of neighborhood effects on health in the U.S. and Europe, including social, environmental, geographical, institutional, and environmental mechanisms. Their review found that neighborhood cohesion can be a protective factor for children, parenting strategies may be influenced by perceptions of their neighborhoods, environmental risks in neighborhoods adversely impact health outcomes, and outside mentors are often brought in to mitigate the effects of fewer or lower-quality child and family service resources and other social programs in under-resourced neighborhoods. More recently, Minh and colleagues’ [41] systematic review of 34 studies on the effects of various neighborhood characteristics on child development showed that neighborhood poverty negatively impacted developmental health. The findings also suggested the importance of looking across groups and settings to more fully understand how neighborhood factors like urbanicity and individual factors such as race might impact children’s experiences within neighborhoods. 

Although previous reviews have considered the impact of neighborhood context on child health, understanding neighborhood poverty and neighborhood gentrification’s unique impacts on child depression, depressive symptoms, and mood disorder prevalence is important for policy and practice. Therefore, the following research question guided this review:

- Is neighborhood poverty/gentrification associated directly or indirectly with the prevalence and symptoms of mood disorders among children and adolescents?

## 2. Materials and Methods

### 2.1. Criteria for Study Selection

Study selection for this scoping review was based on Preferred Reporting Items for Systematic Reviews and Meta-Analyses (PRISMA) standards and the Cochrane Collaboration-approved Population, Intervention, Comparison, and Outcomes (PICO) criteria [42]. PICO criteria were used to loosely guide the title and abstract screening phase and then applied more stringently during the full article review to narrow the studies to those meeting all inclusion criteria. For example, when an abstract was unclear as to what mental health outcomes were measured, the article was included for full screening. 

The target population was children and adolescents ages 3–17 living in the U.S. at the time of the initial study. If the study was longitudinal or retrospective, participants must have been children/adolescents at baseline but could have reached age 18 or older during the study. Additionally, the sample had to either be racially diverse or majority (over 50%) Black, Indigenous, or People of Color. All study designs, including experimental, quasi-experimental, and non-experimental (e.g., observational or correlational) were eligible for inclusion as long as child or adolescent neighborhood poverty and/or gentrification status and depression/mood disorders were measured. In addition, comparative inclusion criteria had to have been applied to the predictor variable, e.g., a measure of neighborhood poverty/gentrification with at least two neighborhoods. The neighborhood poverty/gentrification measure could be categorical/interval (e.g., low-, mid-, and high-income neighborhoods or gentrified and non-gentrified) or continuous (e.g., percentage of people living in poverty in a U.S. census tract). Furthermore, neighborhood poverty/gentrification had to be based on a standardized measure (e.g., Census Bureau Reports) rather than on a child or parent’s perception or beliefs of neighborhood poverty/gentrification. If the study was longitudinal and children/adolescents aged into adulthood during the study period, the initial neighborhood poverty/gentrification variable must have been recorded when participants were children/adolescents. 

The outcome variable had to be a measure based on DSM-IV mood disorders or the DSM-5 distinction of Bipolar and Depressive Disorders, including bipolar and depressive disorders, cyclothymic disorder, major depressive disorder, and dysthymia [43]. Since children were the focus of the scoping review, measures that captured depressive symptoms but not diagnoses were also included because (1) many disorders have a minimum age for diagnosis, and (2) those who lack a history of treatment might also lack an official diagnosis while still having symptoms. 

### 2.2. Search Strategies

**Figure A1:**
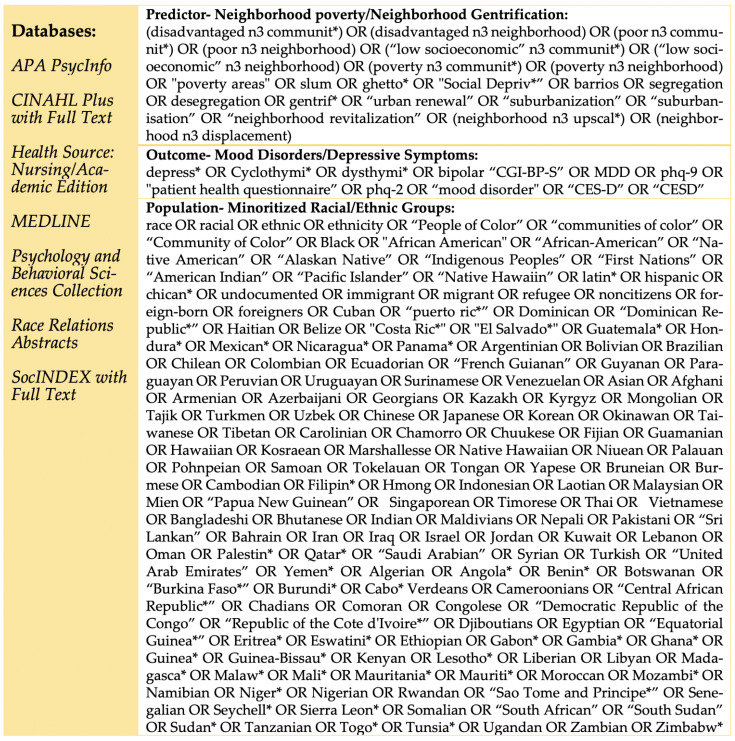
Search terms and database list for scoping review.

To ensure a thorough literature search, a wide range of search terms for neighborhood poverty/gentrification, depressive/bipolar/mood disorders, and racial/ethnic groups were used to scope the following databases: APA PsycInfo, CINAHL Plus with Full Text, Health Source: Nursing/Academic Edition, MEDLINE, Psychology and Behavioral Sciences Collection, Race Relations Abstracts, and SocINDEX with Full Text. Articles had to be written in English or have an English translation available. Dissertations and unpublished reports were included. No date restrictions were applied. Figure A1 contains a full list of the search terms and final database list, which were compiled with the help of a scoping-review-trained social science librarian. Comprehensive search techniques were used (e.g., asterisks to specify that a word can end in several ways) (Figure A1 contains further examples). Additionally, hand-searched articles, grey literature, and seminal articles were included based on Google Scholar searches using similar search terms with fewer quotations and restrictions. 

### 2.3. Data Collection and Analysis

Abstracts and titles from both the database and hand searches were inputted into Rayyan Qatar Computing Research Institute (QCRI) software for systematic and scoping reviews to conduct abstract and title screening, which provided the capacity to assign articles using inclusion and exclusion criteria labels. Articles were included in the initial screening if they named or implied a mental health outcome and a measure of neighborhood poverty/gentrification. Any ambiguity in an abstract resulted in including the article for full screening. Rayyan provides the title, abstract, authors, journal, publication type, keywords, URL, and number of pages for all search results. Articles were excluded in the full-article screening round if they featured adult-only samples (with no longitudinal or retrospective measures of childhood or adolescent neighborhood poverty/gentrification) rather than in the title and abstract screening to avoid eliminating any in which the study population or sample were children/adolescents at the study’s beginning. Full-article screening ensured that the included articles met the rest of the inclusion criteria. Finally, a summary, synthesis, analysis, and critique of the methods, results, and implications were compiled for each included study. 

## 3. Results

The database search, completed on 10 March 2023, resulted in an initial 1519 articles (1073 after removing duplicates), and the hand-search, completed on 1 March 2023, resulted in 17 publications [44,45,46,47,48,49,50,51,52,53,54,55,56,57,58,59,60]. During the abstract/title screening in Rayyan QCRI, 993 articles were excluded, with the primary reasons being that the study was based outside the U.S., there were no neighborhood variables, or mental health variables were predictors rather than outcomes. Less common reasons for exclusion included the lack of a comparison neighborhood (e.g., the study was based in a low-income neighborhood, but no data were presented about those living in higher income neighborhoods), the paper was theoretical and did not include an empirical study, or the study focused on parent rather than child outcomes. 

**Figure A2:**
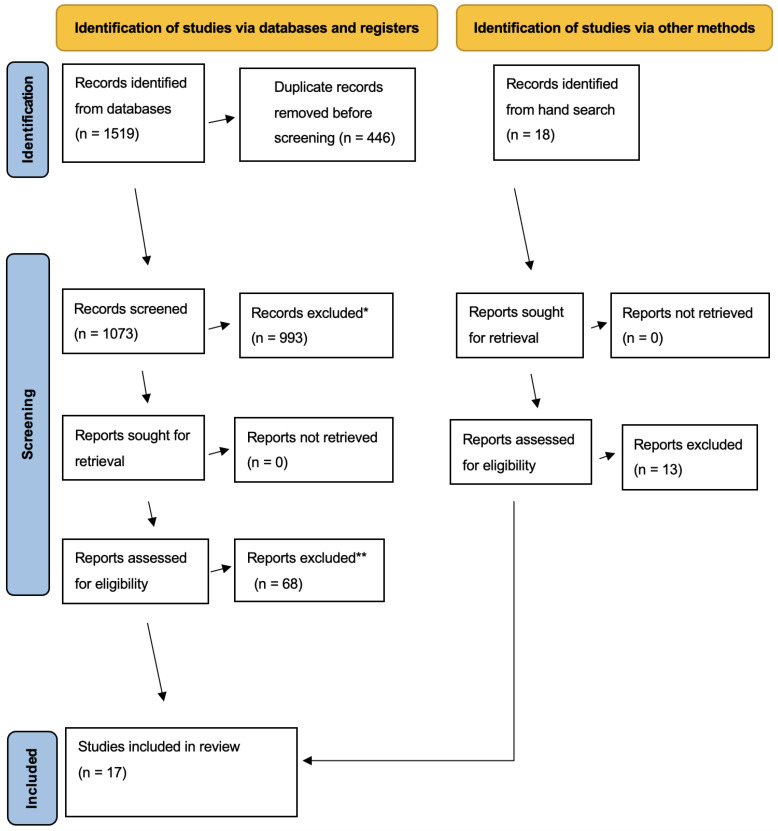
PRISMA flow diagram for scoping review. * The most common reasons for exclusion were a study based outside of the U.S., no neighborhood variables, or mental health variables were the predictor rather than outcome. ** The most common reasons for exclusion were non-children-/adolescent-focused studies, lack of depression or mood disorder in outcomes, and survey respondents’ subjective measures of neighborhood poverty [85].

The included publications from the abstract/title screening and hand-search resulted in 98 publications that were read in full to determine exclusion and inclusion; this full-article review resulted in excluding 81 publications. The final search resulted in 17 included publications published from 1996 to 2019. Fourteen were reported in journal articles, and three were dissertations [47,48,54]. Figure A2 contains the Preferred Reported Items for Systematic Reviews and Meta-Analyses (PRISMA) chart.

### 3.1. Sample

The total sample size of the 17 included studies was 122,089 participants; the largest sample was 71,835 [50], the smallest was 126 [56], and the average sample size was 7134.06. In six studies, participants were adolescents (or were adolescents at baseline in longitudinal studies), eight focused on children (or children at baseline in longitudinal studies), and four included both children and adolescents. The age range for all 17 studies was 3–17 years old (at baseline for longitudinal studies). Seven studies featured longitudinal designs [44,47,49,51,54,58,60], and two featured experimental designs [52,55]. Although longitudinal studies do not rule out confounding effects as well as experimental designs, they do provide important evidence for temporal precedence. Only one study did not utilize hierarchical, multilevel, or other nested modeling [46]. The majority of children and/or adolescents in five study samples were Black [46,51,53,54,57]; in two studies, the majority were Latine [44,54]; in the remaining ten studies, the samples were racially/ethnically diverse. Sixteen studies included female and male children and/or adolescents; one included male children only [55]. No trans or non-binary participants were reported. Although the inclusion criteria required at least two neighborhoods, all included studies featured far more. 

### 3.2. Neighborhood Poverty and Gentrification Measures

Neighborhood measures in the included studies can be divided into three categories: (1) neighborhood poverty indices (based on multiple forms of U.S. census tract data); (2) inventories completed by independent researchers; and 3) experiencing a change in neighborhood income (e.g., moving from a low- to high-income neighborhood during experimental design studies or experiencing gentrification or not). Fourteen studies used neighborhood poverty indices, two measured a change in neighborhood income through gentrification or moving [50,55], and one used an independent inventory of neighborhood poverty [56]. As specified in the exclusion criteria, studies relying on participants’ subjective self-reports of neighborhood poverty/gentrification were not included. 

### 3.3. Depression/Depressive Symptoms 

The depression measures in the 17 studies can be categorized as follows: (1) diagnosed depression disorder according to the DSM-IV or DSM-5; (2) self-endorsed depressive symptoms using a validated scale; (3) parental-endorsed depressive symptoms using a validated scale; and (4) one study did not report using a scale, although it is likely that participants marked if they had ever been diagnosed with depression [50]. The most common scales were the Center for Epidemiologic Studies Depression Scale (CES-D), Composite International Diagnostic Interview (CIDI), Clinical Interview for Depression (CID), Brief Symptom Inventory, Behavior Problem Index, The Child Behavior Checklist (CBCL), and the Reynolds Depression Scale. None of the studies used measures of bipolar disorder, dysthymia, or other mood disorders (many excluded studies measured these disorders but did not meet other inclusion criteria). 

### 3.4. Significance of Results 

Twelve of the seventeen included studies found a significant direct relationship between neighborhood poverty/gentrification and depression measures. Four of these twelve also found a significant mediation or moderation effect (discussed later). The findings were significant in three of the seven studies with child-only participants, five of the six studies with adolescents-only participants, and all four studies with both children and adolescents. 

Although five of the seventeen studies found no significant direct relationship between neighborhood poverty/gentrification on depression measures [44,49,53,56,57], two of these five did find that neighborhood poverty/gentrification significantly mediated or moderated depression [44,57], as discussed later. 

#### 3.4.1. Children

Of the studies with children only, all three longitudinal studies found significant results [44,47,60], while two of the three cross-sectional studies did not find significant results [53,56]. This may indicate delayed effects of neighborhood poverty/gentrification on childhood depression. 

Caughy et al. [46] found that neighborhood impoverishment was significantly related to child depression scales in their cross-sectional analyses. More specifically, children in the lowest-income-quartile neighborhood scored significantly higher on the depression scales than children in other neighborhood quartiles, but in post hoc *t*-tests, no significant differences emerged between neighborhood quartiles. Using U.S. census data, Dearing [47] found that increased neighborhood quality was associated with decreased depression scale scores in the first and second study waves (Wave 1, b = −0.19, *p* < 0.01, at Wave 2: b = −0.31, *p* < 0.01), but not the third. Xue et al. [60] found that a higher concentration of neighborhood disadvantage was associated with higher child/adolescent depression raw scores (b = 0.08, *p* < 0.01) and an increased likelihood of child depression scores meeting a clinical threshold (LO = 0.19, *p* < 0.05) (Table A1 contains all reported details and operationalizations of neighborhood measures). 

#### 3.4.2. Adolescents

In the Moving to Opportunity experiment for adolescent males, Rudolph et al. [55] found that those living in families receiving a housing voucher to move from public housing to a low-income neighborhood in New York City had an increased likelihood of endorsing DSM criteria for Major Depressive Disorder, while those in Chicago had a decreased likelihood. Rudolph suggested that this difference may be due to city-level differences (i.e., housing markets, economies, etc.); therefore, the benefits from the Moving To Opportunity experiment may not be transferrable to every city. Wickrama [58] found that after adding individual and school-level variables to models examining the association between a U.S. census-based-neighborhood concentrated poverty index and adolescents’ depressive symptom scores, the relationship between the two was no longer statistically significant. 

Hurd et al. [51] found that increases in U.S. census block group neighborhood poverty rate were associated with increased depression scores among adolescents. Specifically, the intraclass correlation (ICC) for depressive symptoms between neighborhoods was 5% and the neighborhood poverty rate to social support had a significant indirect effect on depressive symptoms (path a: (b = −1.02., *p* < 0.05) and path b: (b = −0.32, *p* < 0.05)). Additionally, neighborhood unemployment rate to perceived neighborhood cohesion had a significant indirect effect on depressive symptoms (path a: (b = −0.48, *p* < 0.05) and path b: (b = −0.20, *p* < 0.05)). Finally, Aneshensel and Sucoff [45] found that Latine youth living in neighborhoods with a low socioeconomic (SES) index had lower depression scores (b = −0.418, SE = 0.17, *p* < 0.05). This is the only included study where an increase in neighborhood poverty was associated with a decrease in the depression outcome. However, Aneshensel et al. [45] noted that Latine youth consistently had higher rates of depression than other racial/ethnic groups, but those living in neighborhoods with a high concentration of Latine residents had a lower rate of depression. The authors, therefore, pointed to the importance of measuring neighborhood racial composition in addition to neighborhood poverty (see Table A2 for all reported details).

#### 3.4.3. Children and Adolescents

Leventhal and Brooks-Gunn [52] found that children aged 8–13 years old who moved to less impoverished neighborhoods reported significantly fewer anxiety/depressive symptoms. More specifically, children who moved reported lower anxiety/depression compared to children who remained in low-income neighborhoods (b = −0.39, SE = 0.21, *p* < 0.05) and compared to children living in Section 8 Housing (b = −0.90, SE = 0.49, *p* < 0.05). Leventhal and Brooks-Gunn [52] also found significant relationships for male but not female children: boys who moved (experimental group) had higher anxiety/depression compared to boys who remained in low-income neighborhoods (b = −0.42, SE = 0.21, *p* < 0.01) and compared to those living in Section 8 housing (b = −1.20, SE = 0.65, *p* < 0.05). 

Delany-Brumsey [48] found that increased neighborhood disadvantage was associated with increased child/adolescent depression scores (b = 0.51, SE = 0.16, *p* < 0.01). Dragan et al. [50] found that living in gentrifying neighborhoods was associated with increased DSM diagnoses of child and adolescent depression compared to those who remained in low-SES neighborhoods. More specifically, the rate of depression diagnoses in children living in gentrified neighborhoods was 22% higher than for children living in low-income neighborhoods. While all other health problems decreased on average, depression diagnoses were the only problems that worsened in gentrifying areas. The authors suggested that perhaps this is because low-income families who stay in gentrifying areas face increased economic pressure. This claim was also supported given that this effect was present for children living in market price housing where rent increased with neighborhood gentrification but not for children living in subsidized housing. Patrick [54] found that increases in a U.S. census-based-neighborhood concentrated poverty index was associated with an increased likelihood of depression/anxiety symptoms among all adolescent participants (b = 1.06, SE = 0.48, *p* < 0.05), and for children, among male (b = 1.28, SE = 0.56, *p* < 0.05) but not female participants (see Table A3 for all reported details and operationalizations of neighborhood measures). 

### 3.5. Significant Mediation/Moderation

In four studies, neighborhood poverty significantly mediated or moderated the relationship between a predictor and the depression measure. In Alegria et al. [44], neighborhood poverty significantly mediated the relationship between child ethnicity and depression. In Simons et al. [57], neighborhood poverty significantly moderated the relationship between neighborhood criminal victimization and child depression; those in low-income neighborhoods had more depressive symptoms due to criminal victimization than those in high-income neighborhoods. In Caughy et al. [46], neighborhood poverty moderated the relationship between maternal social capital and child depression; there was a stronger relationship between maternal social capital and child depression in low poverty neighborhoods compared to high poverty neighborhoods. Finally, in Wickrama et al. [58], neighborhood poverty moderated the relationship between race and adolescent depression; specifically, the relationship between neighborhood poverty and depression was significant and stronger for Asian and African American youth than white youth.

## 4. Discussion

In 70% of studies (12 of 17) included in this scoping review, neighborhood poverty/gentrification directly predicted depression outcomes, and when included as a mediator or moderator, neighborhood poverty/gentrification directly or indirectly predicted depression outcomes in 82% (14 of 17) of the studies. In 93% (13 of 14) of the significant direct/indirect predictor studies, neighborhood poverty/gentrification was associated with increased depression. In studies of children, the most common depression measure was the CBLC [46,53,60]; in studies of adolescents, it was the CES-D [49,58,59]; and in studies including both children and adolescents, it was the Behavior Problem Index [48,52]. Studies that used the same depression measure tended to find similar results; for example, both Li et al. [53] and Schaefer-McDaniel et al. [56] found no significant associations between neighborhood poverty/gentrification and depression using the CDI. 

Fourteen studies controlled for family or individual income, while two studies controlled for living in public housing and receiving other public assistance as a measure of economic distress [50,55]. Alegria et al. [44] was the only study that did not report an individual income control. Overall, more studies showed significant relationships between neighborhood poverty/gentrification and depression when samples included or were solely composed of non-Latine Black children or featured diverse samples. Two studies reported similar gender disparities: both Patrick [54] and Leventhal et al. [52] found significant relationships between neighborhood poverty/gentrification and depression measures for male, but not female, children. Finally, in the studies with samples that contained children only, just four of the seven neighborhood poverty/gentrification measures were significant direct predictors of childhood depression. However, due to the small sample sizes of included studies, these observational data should not be used to generalize about the age groups. Additionally, this phenomenon may be due to delayed development of depression, i.e., neighborhood poverty/gentrification may have a lagged effect with depression developing later in life, or perhaps because as children grow older, they become more aware of neighborhood income disparities. 

### 4.1. Methodological Basis 

#### 4.1.1. Neighborhood Measures

Although all the included studies are bolstered by their objective rather than subjective self-reported measures of neighborhood poverty/gentrification, some measures had serious limitations. The objective indices were based on U.S. census data, or in one case, researcher ratings of neighborhoods [56]; however, the choice of census data indicator may not have captured neighborhood-level poverty/gentrification entirely. Both Li et al. [53] and Simons et al. [57] used only U.S. census block group information to identify the percent of people living below the poverty line as a measure of neighborhood poverty, a conservative estimate of poverty’s prevalence. Other studies used the percentage of female-headed households or percentage of Black and/or Latine residents in a neighborhood as a part of their index. Although these decisions were usually based on prior research indicating that poverty is higher among these groups than among whites, neighborhood racial composition is not necessarily a measure of poverty and may make it difficult to measure the collective efficacy often found within communities of color that can serve as a protective factor for mental health. For example, Aneshenesel et al. [45] found that controlling for neighborhood racial composition and neighborhood poverty, living in a majority Latine neighborhood protected Latine youth against depressive symptoms. In addition, most studies used census block group data on poverty; although this is perhaps the most readily available objective measure, census block group data may not capture the area that residents consider to be their neighborhood. 

Few studies addressed urban planning factors (e.g., a history of redlining) or urban design factors (e.g., prevalence of parks or libraries). Schaefer-McDaniel [56] was the only study to include a measure of neighborhood poverty that addressed the state of buildings and other urban design factors. The lack of these types of neighborhood indicators may impede the accurate measurement of neighborhood residents’ experiences. Finally, another important criticism of neighborhood poverty measures is that they generally focus on measuring disadvantages. A 2011 Urban Institute paper recommended including neighborhood assets (e.g., job density) and community resources or opportunities in neighborhood SES measures [43], as they may be protective factors.

#### 4.1.2. Depression Measures 

Many of the studies used self-reported diagnoses or depressive symptoms, which are subject to overreporting or underreporting bias [61]. They may also be less reliable than validated depression scales (e.g., the CES-D). 

#### 4.1.3. Statistical Analyses

Most studies used appropriate complex statistical models. Hierarchical linear modeling or experimental analyses are imperative for analyzing nested data to account for the shared variance of individuals (i.e., children/adolescents) within the same groups (i.e., neighborhoods). Only one study used a questionable analytical approach, i.e., conventional regression analysis, where statistical assumptions would be violated using this approach with nested data, although preliminary ANOVA tests were conducted [46]. In addition, several studies that used hierarchical, multilevel, or nested data analysis techniques did not report ICC to indicate how much variance in the depression outcome lies between neighborhoods. 

### 4.2. Theoretical Basis 

Neighborhood poverty/gentrification functioned as a social determinant of depressive symptoms in most of the included studies. Cutrona et al. [34] offered a conceptual framework identifying several neighborhood characteristics that increased stress in their study sample, leading to increased depression: (1) a lack of resources increasing residents’ daily stress; (2) low-income neighborhoods compounding repercussions of the first characteristic (i.e., depression may be more likely in someone experiencing job loss in a low-income compared to a high-income neighborhood) [38]; and (3) high resident turnover in low-income neighborhoods leading to higher rates of depression [34]. Neighborhood poverty perhaps signified a lack of resources for children and adolescents in this review, increasing depressive symptoms. 

#### 4.2.1. Life-Course Theory

Life-course theory is a primary theory that can be used to explain how early childhood experiences and environments shape health outcomes throughout life [62]. Only Wickrama’s [58] study specifically mentioned life-course theory principles (i.e., life events) as a guiding framework. However, eleven studies appeared to support the theory despite not naming it, as they found direct relationships between child or adolescent neighborhood poverty/gentrification and measures of depression over different life stages. Partially supporting life-course theory, Dearing [47] found a short-term effect of neighborhood poverty on children’s depression (i.e., at study waves one and two but not three). This may indicate that neighborhood poverty affects childhood depression only in the short term, though effects later in life cannot be ruled out. 

Further supporting the effects of early childhood experiences, Delany-Brumsey [48] found that neighborhood poverty significantly affected depression in children but not adolescents. Other factors (e.g., peers and schools) may be more important for adolescents’ mental health than neighborhood effects. To further test life-course theory, neighborhood poverty/gentrification could be measured throughout life to account for changing income levels in neighborhoods along with contemporaneous measures of depression. Longitudinal or retrospective data are a prerequisite to understanding lifespan effects, and no included study examined waves of data from childhood to adulthood. 

#### 4.2.2. Ecological Systems Model and Ecosocial Theory 

Two studies [45,51] referenced the ecological systems model, which posits that understanding a person’s growth requires understanding the systems in which that person matures [63]. All 14 studies with significant findings seem to support the ecological systems model, as they reported associations between neighborhood poverty/gentrification and individuals’ depression. Determining neighborhood effect can be difficult when building these types of models because it is possible to overcontrol the path from neighborhood characteristics to an outcome.

Only Dragan et al. [50] addressed how neighborhood gentrification altered the system around the child. Despite increased neighborhood median income due to neighborhood gentrification, children living in gentrified areas were more likely to report depression than those living in persistently low socioeconomic areas. In contrast, experimental design studies, in which children (and their families) were moved to another neighborhood, demonstrated some favorable results in depression risk reduction [52]. A criticism of the ecological systems model is that it often emphasizes individual and family characteristics rather than neighborhood and structural factors. Many included studies focused on individuals’ perceptions of neighborhood safety or their neighborhood social connections and included neighborhood poverty only as a control variable/correlate, despite neighborhood poverty/gentrification being associated with depression. Framing neighborhoods in terms of individual-level factors may obscure the larger structural forces that determine mental and physical health. 

Ecosocial theory builds on the ecosystems model and may add what the ecological model lacks—a lifespan perspective, historical context (e.g., redlining), social factors (e.g., structural racism, sexism, and homophobia), and biology. Ecosocial theory also requires researchers to study social disparities in health [64]. The majority of included studies met this latter charge because they studied neighborhoods’ role in mental health disparities. However, no study explicitly mentioned ecosocial theory or its components. Although a few studies considered how racial/ethnic identity (often used as a proxy for racism) impacted the neighborhood poverty/gentrification–depression relationship [44,57,58], none specifically investigated racism or other forms of discrimination at the individual and/or community level. 

### 4.3. Implications

#### 4.3.1. Policy

This review informs policymakers of the importance of reducing neighborhood poverty/gentrification as a means of preventing mental health problems among children and adolescents in the U.S. First, policies that decrease economic inequality will likely be tied to decreases in neighborhood-level poverty, which, in turn, will likely reduce the associated risk of depression. Programmatic efforts such as cash benefits, improving the healthcare infrastructure (e.g., through Medicaid expansion), increasing earned income tax credits for low-income families, and providing a living wage are all policy initiatives that can mitigate individual- and neighborhood-level poverty. Increasing school funding in low-income neighborhoods, the number of job opportunities through in-person or remote work, and legal protections for workers’ unions have all been associated with decreases in individual poverty [65,66,67]. Furthermore, by highlighting the impact of neighborhood poverty on depression, this scoping review informs policymakers that poverty reduction as a form of investment in community mental health might be a promising strategy. For example, a UK study reported that introducing a livable minimum wage had practically the same effect on depression reduction as antidepressants [68]. 

Reducing the racial wealth gap would impact individual- and neighborhood-level poverty. This review further highlights the importance of reducing this gap since all included studies consisted of racially/ethnically diverse populations. Non-Latine white families have 18 times the wealth of non-Latine Black families and seven times the wealth of Latine families [69]. Policies and programs to assist families in asset building, particularly in homeownership, as it alone accounts for 62% of families’ wealth [70], may decrease depression among Black and Latine children. 

Housing reparations may also provide opportunities to reduce neighborhood poverty when education, marital status, and work opportunity do not. Reparations are “a system of redress for egregious injustices” [71], such as the USD 1.5 billion paid to Japanese Americans who survived internment during WWII [72]. Housing reparations to disinvested Black and Brown communities, particularly those with a history of historical redlining, could help reduce neighborhood poverty and wealth inequality [73]. The scoping review also demonstrated that gentrification can adversely impact child and adolescent depression. Therefore, preventing resident displacement in neighborhoods at risk of neighborhood gentrification through constructing more affordable housing units, retention of affordable housing (e.g., publicly subsidized rental housing), programmatic asset-building support for residents (e.g., job training), and rental caps/rent control may prevent displacement [73], thereby improving mental health. 

Neighborhood revitalization programs, which provide funding for building greenspaces, parks, libraries, or communal spaces, and updating neighborhood and school infrastructure may also decrease the impact of neighborhood poverty by improving neighborhood quality of life. Gariepy et al. [74] reported that living near a park or local health services decreased the risk of adult depression. Holzer [75] found increased neighborhood wealth after low-income neighborhoods received USD 400 million through the Minneapolis Neighborhood Revitalization Program. 

#### 4.3.2. Clinical Services

Clinicians who provide physical and behavioral health care also have a role in advocating for policies such as parity to reduce barriers to mental health care [76]. The earlier mental health interventions are provided, the better children’s outcomes are, but children of color residing in low-income neighborhoods are less likely to have access to or receive effective mental health services [77]. Mental health workforce shortages are a significant contributor to the glaring inequities in mental health care. The Health Resources and Services Administration funds training programs to reduce these shortages, attract trainees from diverse backgrounds, and better prepare the behavioral health workforce to serve historically underserved communities. Clinicians can also advocate for improved housing and other resources for low-income clients that enhance mental and physical well-being. 

#### 4.3.3. Research

Despite significant research on neighborhood environments as a social determinant of physical health, less research has been conducted on the neighborhood as a social determinant of mental health. Researchers should address the methodological and theoretical critiques presented in this scoping review and continue to investigate relationships between neighborhood poverty and mental health outcomes. Study designs should continue to include multiple indicators to capture the latent concept of community poverty, such as using average neighborhood property values [78], average rents, the number of evictions and foreclosures [79], and local school quality [80] in addition to poverty data available from the U.S. census. Researchers can also strengthen neighborhood measures by including other indicators of neighborhood poverty/gentrification such as urban design and urban planning features. For example, neighborhood quality can be rated using highly detailed virtual maps to observe building conditions and prevalence of businesses.

Additionally, researchers can continue measuring different facets of life in low-income neighborhoods, such as the length of time children and adolescents spent in a neighborhood, their age when they lived in a particular neighborhood, changes in neighborhoods, and moving to or from low-income neighborhoods. More quasi-experimental studies are needed to better determine causality between neighborhood context and mental health outcomes. In particular, researchers should investigate childhood depression due to neighborhood poverty in longitudinal studies, since the environment may have a lagged effect on health [81]. 

Neighborhood gentrification, which has been studied less than neighborhood poverty, deserves particular focus as a social determinant of depression. To study neighborhood gentrification, measures like Bilal et al.’s [82] 16-item measure of neighborhood economic change should be utilized. Additionally, researchers should include measures of neighborhood racial and ethnic composition, preferably as a measure separate from neighborhood poverty. Researchers should use validated scales to measure depression, dysthymia, and other mood disorders such as the CES-D for adolescents [83] and the Child Behavior Checklist for children [84]. To broaden the scope of this review, researchers should examine additional mood disorder outcomes.

The study methodology should account for the nested nature of neighborhood data by using appropriate multilevel modeling techniques and by reporting important statistics (e.g., ICC and model fit). Researchers should also continue to test mediation and moderation pathways where the neighborhood is involved and include mezzo- and macro-level variables rather than individual level-variables exclusively. Finally, researchers should increase attention to ecosocial theory and other comprehensive theoretical frameworks [64]. 

## 5. Conclusions

Neighborhood poverty and neighborhood gentrification in the U.S. impact depression and depressive symptoms among children and adolescents. This review underscores the importance of continuing to study neighborhood characteristics as determinants of mental health, as most included studies indicated a correlation between neighborhood poverty/gentrification and child depression.

Based on the findings, mental health training and educational programs should persist in highlighting the importance of neighborhood history and the ongoing socioeconomic and racial segregation prevalent in societies today. Researchers should continue examining this issue, particularly by using longitudinal and quasi-experimental design studies. Neighborhood poverty and gentrification require further examination as social determinants of other mental health outcomes (e.g., anxiety) given their impact on child depressive symptoms. Practitioners and policymakers should consider ways to address economic inequality among gentrifying and low-income neighborhoods. Studying neighborhood effects as social determinants of mental health is a social justice imperative that will support policy changes to mitigate inequities in children’s mental health. 

## Data Availability

All studies included in this review are cited in the references below.

## Appendix A

**Table A1:** Results for included studies with child samples organized by study design.

First Author	Year	Sample Size	Data Source and Population ^£^	Study Design	Neighborhood Poverty or Gentrification	Depression Outcome ^†^	Results	Coefficients and Estimates
Alegria	2019	2491	Boricua Youth Study Puerto Rican children (5-13 years old)	Longitudinal	Neighborhood context: Proportion of people living below the poverty line, percentage of female-led households, and percentage of Latine households	CIDI (with self-reported Major Depressive Disorder) and DAS	Neighborhood poverty indicators mediated the relationship between minority status and Major Depressive Disorder diagnosis. Neighborhood poverty indicators were not included in the full model.	Proportion of female-headed households with child under 18 significantly mediated (b = 0.22, SE = 0.01, *p* < 0.001) the relationship between minority status and mental health outcomes.
Dearing ^‡^	2001	206	Data from four Boston elementary schools Children (7-8 years old)	Longitudinal	Neighborhood Quality: 1990 Census neighborhood percentage of people living in poverty and reported crime rate for that neighborhood	Reynold’s Depression Scale	Increases in neighborhood quality led to decreases in childhood depression in waves one and two but not three.	Wave 1 (b = -0.19, SE = NR, *p* < 0.01) Wave 2 (b = −0.31, SE=NR, *p* < 0.01) Wave 3 non-significant
Xue	2005	2805	Project on Human Development in Chicago Neighborhoods Children (5-11 years old)	Longitudinal	Concentrated neighborhood disadvantage: Neighborhood poverty rate, percentage of residents receiving public assistance, percentage of female-headed families, unemployment ratio, and percentage of African American residents	CBLC/4-18 and then an additional dichotomized clinical threshold variable	Concentrated disadvantage was associated with more mental health problems and a higher number of children in the clinical range. Collective efficacy mediated the effect of concentrated disadvantage. Once collective efficacy was added to the model, concentrated disadvantage was no longer directly significant.	ICC= 11.1%. Concentrated disadvantage for total raw score (b = 0.08, SE = 0.03, *p* < 0.01) and dichotomous clinical threshold (LO = 0.19, SE = 0.09, *p* < 0.05)
Caughy	2003	200	Data from Baltimore urban center Black children (3-4 years old)	Cross-sectional	Neighborhood Impoverishment: Neighborhood poverty rate, unemployment rate, vacant housing rate	CBLC/2-3 and CBLC/4-18	Low-income neighborhoods were associated with higher internalizing problems among children compared to other quartile neighborhoods. Rate of total problem behaviors and internalizing behaviors for children living in neighborhoods in the lowest quartile of impoverishment was higher if their mothers knew very few neighbors.	Lowest income quartile neighborhood (F = 3.57, DF = NR, *p* < 0.05) and mothers’ high social capital significantly interacted with/strengthened the relationship between neighborhood income and child internalizing behaviors
Li	2007	263	Data on eight Chicago elementary schools Children (8-9 years old)	Cross-sectional	Neighborhood Poverty: Census block groups	Average of CDI, HIF, and CBLC parent report form	Relationship between neighborhood poverty and children’s internalizing symptoms was not significant.	Non-significant
Schaefer-McDaniel	2009	126	Data on three New York neighborhoods Children	Cross-sectional	Neighborhood Social Disorder: Systematic Social Observations inventory with two independent raters	CDI	No significant direct relationship between neighborhood social disorder and childhood depression, but child subjective evaluations of neighborhood quality fully mediated the relationship between drug/alcohol stressors and childhood depression.	Non-significant
Simons	2002	867	Data from Iowa and Georgia Family and Community Health Study Black children (10–12 years old)	Cross-sectional	Neighborhood Poverty: Census block data on proportion of households below the poverty line in each block group	DISC-IV	No significant direct relationship between neighborhood poverty and child depressive symptoms, but neighborhood poverty significantly moderated the relationship between being the victim of a crime and child depressive symptoms.	ICC = NR Living in a high poverty neighborhood significantly interacted with/strengthened the relationship between being the victim of a crime and child depressive symptoms (b = −0.24, SE = NR, *p* < 0.05)

**Notes:** ^†^ Labelled as depression outcomes since none of the included studies reported measures of bipolar disorder, dysthymia, or other mood disorders; **^£^** if the sample featured only one or two racial groups, the racial composition of the sample was noted; ^‡^ indicates a dissertation rather than a journal article. **Key:** NR = not reported in the study; ICC = intraclass correlation at the neighborhood level; Non-significant = not significant at *p* < 0.05 level; SES = socioeconomic status; CES-D = Center for Epidemiologic Studies Depression Scale; CIDI = Composite International Diagnostic Interview; CID = Clinical Interview for Depression; CBLC = The Child Behavior Checklist; HIF = How I Feel Scale; DISC-IV = Diagnostic Interview Schedule for Children.

**Table A2:** Results for included studies with adolescent samples organized by experimental, longitudinal, and cross-sectional designs.

First Author	Year	Sample Size	Data Source and Population ^£^	Study Design	Neighborhood Poverty or Gentrification	Depression Outcome ^†^	Results	Coefficients and Estimates
Rudolph	2017	1094	Moving to Opportunity data Black and Latino adolescent males	Experimental ^⸹^	Moving from a low-poverty neighborhood: Residents living in high poverty census tracts within five U.S. cities were randomly allocated housing vouchers to relocate away from high poverty neighborhoods.	Major Depressive Disorder according to DSM	Cities had similar rates of depression and moving did not give definitive results.	Housing voucher receipt increased risk of major depressive disorder among boys in New York City but decreased risk in Chicago (*p*-value for site difference = 0.10)
Donnelly	2016	2264	The Fragile Families and Child Wellbeing Study Adolescents (born 1998–2000)	Longitudinal	Neighborhood SES: Census data on percentages of residents who were poor, unemployed, and receiving public assistance and of residents without a high school diploma and with a bachelor’s degree; the percentage of workers with professional or managerial occupations; and percentage of households headed by a female	CES-D	Non-significant, but neighborhood collective efficacy improved mental health.	Non-significant
Hurd	2013	571	Sample from four high-schools in a midwestern state Black adolescents	Longitudinal	Neighborhood Poverty Rate: Census block group on percent of African American residents, householders living in the same house for over 5 years, families living below the poverty line (poverty rate), and residents over the age of 16 unemployed	Part of the Brief Symptom Inventory	Adolescents in more impoverished neighborhoods reported lower total levels of social support. Lower levels of social support mediated the relationship between neighborhood poverty and depressive symptoms. Neighborhoods with a higher unemployment rate were associated with lower perceptions of neighborhood cohesion.	ICC = 5% Neighborhood poverty rate to social support had a significant indirect effect on depressive symptoms path a: (b = −1.02, SE = 0.45, *p* < 0.05) and path b: (b = −0.32, SE = 0.14, *p* < 0.05) Neighborhood unemployment to perceived neighborhood cohesion had a significant indirect effect on depressive symptoms, path a: (b = −0.48, SE = 0.22, *p* < 0.05) path b: (b = −0.20, SE = 0.09, *p* < 0.05)
Wickrama	2005	20,745	National Longitudinal Study of Adolescent Health (Add Health) Adolescents	Longitudinal	Poverty Concentration Index: Comprised of the proportion of families living in poverty, single-parent families, adults employed in service occupations, and unemployed males	CES-D	Only the interactions between race and community disadvantage were significantly related to CES-D scores among adolescents.	ICC = 14% Significant interactions between race and community disadvantage on CES-D
Aneshensel	1996	877	Data from Los Angeles Neighborhoods Adolescents	Cross-sectional	Neighborhood SES Index	CDI	Latine adolescents in low-income neighborhoods had higher depression CDI scores, except when they lived in neighborhoods with a high concentration of Latine populations.	Majority ethnicity Latine, low-income neighborhoods (b = −0.418, SE = 0.17, *p* < 0.05) Other neighborhoods non-significant
Wickrama	2003	14,500	National Longitudinal Study of Adolescent Health (Add Health) Adolescents	Cross-sectional	Community poverty concentration: Census tract level proportion of families living in poverty, single-parent families, adults employed in service occupations, and proportion of unemployed men	CES-D	Without individual variables, concentration of poverty strongly predicted adolescent depressive symptoms, but after adding individual and school characteristics, it was no longer significant. The sample was also split into “less adverse” and “extreme adverse communities”; in less adverse communities, concentration of poverty predicted depressive symptoms.	ICC = NR Model 1(b = 2.35, SE = NR, *p* < 0.001), Model 2 (b = 0.62, SE = NR, *p* < 0.05) Models 3–5: Non-significant Concentration of poverty significant in less adverse communities (b = 5.18, SE = NR, *p* < 0.05) but non-significant in extreme adverse communities

**Notes:** ^†^ Labelled as depression outcomes since none of the included studies reported measures of bipolar disorder, dysthymia, or other mood disorders; ^⸹^ experimental designs are inherently longitudinal and were therefore not labeled as such; **^£^** if the sample featured only one or two racial groups, the racial composition of the sample was noted; **Key:** NR = not reported in the multilevel study; ICC = intraclass correlation at the neighborhood level; Non-significant = not significant at *p* < 0.05 level; SES = socioeconomic status; DSM = Diagnostic and Statistical Manual of Mental Disorders; CES-D = Center for Epidemiologic Studies Depression Scale; CID = Clinical Interview for Depression.

**Table A3:** Results for included studies with child/adolescent samples organized by experimental, longitudinal, and cross-sectional designs.

First Author	Year	Sample Size	Data Source and Population ^£^	Study Design	Neighborhood Poverty or Gentrification	Depression Outcome ^†^	Results	Coefficients and Estimates
Leventhal	2003	512	Re-interview of the Moving to Opportunity participants Children and Adolescents (8–18 years old)	Experimental ^⸹^	Neighborhood Poverty: Census block data on poverty concentration (comprised of median incomes, fraction of residents in poverty, and fraction of rental units)	Behavioral problem index (anxious/ depressive scales)	Boys who moved to less poor neighborhoods reported significantly fewer anxious/depressive and dependency problems than did boys who stayed in low-income neighborhoods and boys in public housing. Children aged 8–13 reported significantly fewer anxious/depressive symptoms compared to children who stayed in low-income neighborhoods and children in Section 8 Housing.	Boys who moved (experimental group) in comparison to families who stayed in low-income neighborhoods (b = −0.42, SE = 0.21, *p* < 0.01) and in comparison to families in Section 8 housing (b = −1.20, SE = 0.65, *p* < 0.05) Children 8–13 years old in families that moved compared to families that stayed in low-income neighborhoods (b = −0.39, SE = 0.21, *p* < 0.05) and compared to families in Section 8 Housing (b = −0.90, SE = 0.49, *p* < 0.05)
Patrick ^‡^	2019	618	Project on Human Development Chicago Neighborhoods (PHDCN) Black and Latine children and adolescents	Longitudinal	Neighborhood Concentrated Poverty Index: The percentage of poor families, percentage of single parent families, percentage of families receiving public assistance, and unemployment rate	Self-endorsed depressive/ anxiety symptoms	Increases in concentrated poverty significantly predicted self-endorsed depression/anxiety symptoms among all adolescents and male children in one model, but the latter relationship was not significant for male children once other individual factors (i.e., discrimination) were added.	For adolescents (b = 1.06, SE = 0.48, *p* < 0.05). For male children in one model (b = 1.28, SE = 0.56, *p* < 0.05) but non-significant for female children.
Delany-Brumsey ^‡^	2012	1305	Los Angeles Family and Neighborhood Survey (L.A. FANS) Children and Adolescents	Cross-sectional	Neighborhood SES Disadvantage: Percentage of population in poverty, families with an annual income less than $24,000, households headed by females with children, households receiving public assistance, non-White and non-Asian and Pacific Islander, and under age 18	Behavioral problem index (including anxiety and depression subscales)	The level of neighborhood disadvantage was associated with children’s behavioral problems, but it was not significantly associated with either internalizing or externalizing behavior problems in adolescents.	ICC = 12% Disadvantaged neighborhoods (b = 0.51, SE = 0.16, *p* < 0.01). When maternal depression interaction term is included in the model, greater neighborhood economic disadvantage is associated with more behavior problems for adolescents internalizing (b = 0.46, *p* < 0.05).
Dragan	2019	71,835	New York Medicaid Data Set Children and Adolescents (born 2006–2008)	Cross-sectional	Gentrifying Neighborhoods: Bottom 40% of city tract neighborhoods that experienced growth in waves subsequent to baseline	Depression/ anxiety: Not stated, but likely was a self-reported diagnosis	There was a significant increase in depression or anxiety among adolescents in a rapidly gentrifying neighborhood compared to those that remained in low SES areas	Children in areas that gentrified had a 22% higher prevalence of depression/anxiety than children in low-SES areas. There was a 1.56% increase (8.69% versus 7.13%) in depression/anxiety in rapidly gentrifying areas compared to those who remained in low SES neighborhoods

**Notes:** ^†^ Labelled as depression outcomes sine none of the included studies had measures of bipolar disorder, dysthymia, or other mood disorders; ^⸹^ Experimental designs are inherently longitudinal and were therefore not labeled as such; **^£^** If the sample featured only one or two racial groups, the racial composition of the sample was noted; ^‡^ indicates a dissertation, rather than a journal article; **Key:** NR = not reported in the study; ICC = Intraclass correlation at the neighborhood level; Non-significant = not significant at *p* < 0.05 level; SES = socioeconomic status.
